# Supplementary material for: Ribosomal protein S7 ubiquitination during ER stress in yeast is associated with selective mRNA translation and stress outcome
Source: Sci Rep. 2020 Nov 12;10:19669. doi: 10.1038/s41598-020-76239-3 (PMC7661504; doi:10.1038/s41598-020-76239-3)
Supplement: Supplementary file 1 — Supplementary Information 1. [file 41598_2020_76239_MOESM1_ESM.docx]

**Supplemental Information**

**Ribosomal protein S7 ubiquitination during ER stress in yeast is associated with selective mRNA translation and stress outcome**

Yasuko Matsuki^1,7^, Yoshitaka Matsuo^1,7^, Yu Nakano^1^, Shintaro Iwasaki^2,3,4^, Hideyuki Yoko^1^, Tsuyoshi Udagawa^1^, Sihan Li^1^, Yasushi Saeki^5^, Tohru Yoshihisa^6^, Keiji Tanaka^5^, Nicholas T. Ingolia^2^ and Toshifumi Inada^1,*^

**Affiliations:**

^1^Graduate School of Pharmaceutical Sciences, Tohoku University, Sendai 980-8578, Japan.

^2^Department of Molecular and Cell Biology, University of California, Berkeley, CA94720, United States.

^3^Department of Computational Biology and Medical Sciences, Graduate School of Frontier Sciences, The University of Tokyo, Kashiwa, Chiba 277-8561, Japan

^4^RNA Systems Biochemistry Laboratory, RIKEN Cluster for Pioneering Research, Wako, Saitama 351-0198, Japan

^5^Laboratory of Protein Metabolism, Tokyo Metropolitan Institute of Medical Science, Setagaya-ku, Tokyo 156-8506, Japan.

^6^Graduate School of Life Science, University of Hyogo, Hyogo 678-1297, Japan.

^7^These authors contributed equally.

**Supplemental Files**

**Supplemental Figures 1-11**

**
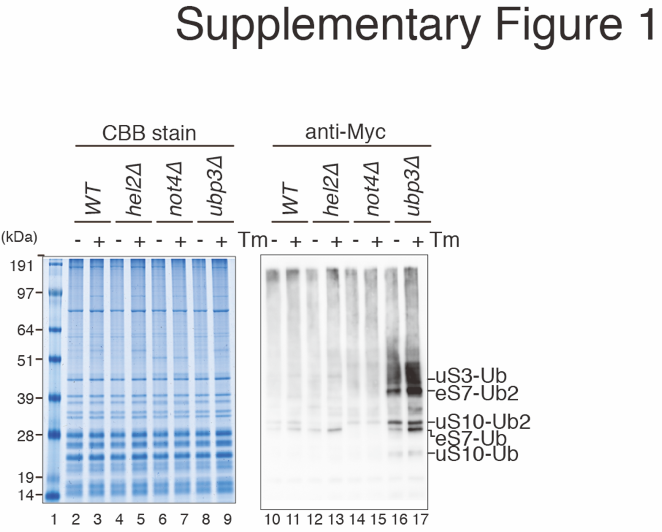
**

**Figure S1. eS7 ubiquitination upon tunicamycin treatment is dependent on Not4.** WT, *hel2*Δ, *not4*Δ, and *ubp3*Δ cells harbouring p*CUP1*p-*MYC-UBI* and p*RPS2(uS5)-FLAG* or p*RPL25(uL23)-FLAG* were cultured in 800 mL of synthetic complete medium. To induce the expression of Myc-Ubi, the cells were cultured in the presence of 0.1 mM Cu^2+^ for 2 h. Cell lysates were prepared and FLAG-tagged ribosomes were purified using M2 FLAG-affinity resin (Sigma), as described (1). Affinity purified samples were subjected to SDS-PAGE followed by western blotting with an anti-Myc antibody. The sections of the gels corresponding to the bands detected by western blotting were isolated and analyzed by mass spectrometry(2).

**
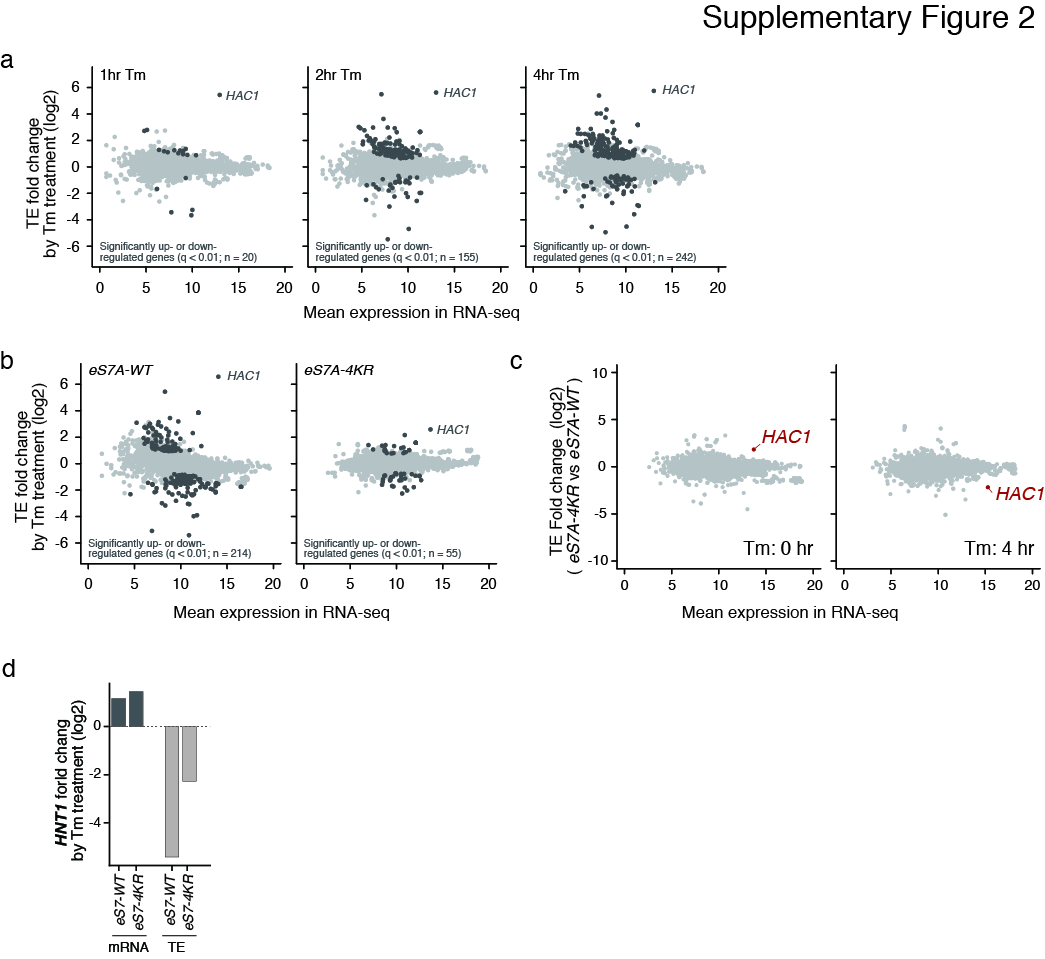
**

**Figure S2. Differential translation efficiency of *eS7A-WT* and *eS7A-4KR* mutant cells in response to Tm treatment.**

**a**, TE change after Tm treatment in WT cells. Wild type cells were treated with 1 µg/mL Tm, and harvested at 0, 1, 2 and 4 h. TE fold changes at the indicated time points relative to 0 h were calculated using Generalized Linear Models in the DESeq package. Genes with q-values <0.01, as calculated by the Benjamini-Hochberg method, are indicated in dark grey. **b**, TE changes in *eS7A-WT* and *eS7A-4KR* cells after treatment with Tm (1 µg/mL) for 4 hr. Genes with q-values <0.01, as calculated by the Benjamini-Hochberg method, are indicated in dark grey. **c**, Translational efficiency in *eS7A-4KR* and *eS7A-WT* cells before and after Tm treatment. TE fold changes were calculated using Generalized Linear Models in the DESeq package. The red dot indicates the TE of *HAC1* mRNA**. d**, Downregulation of *HNT1* translation in response to UPR was diminished in *eS7A-4KR* mutant cells.

**
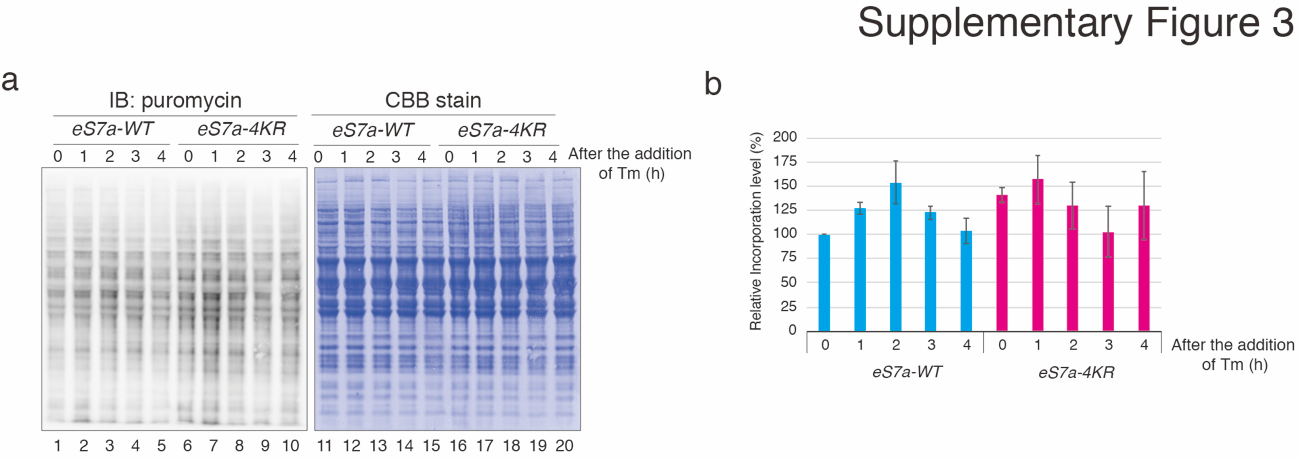
**

**Figure S3. Protein synthesis rates in eS7A-WT and eS7A-4KR cells, as determined by puromycin labelling. a,** Levels of bulk translation in eS7A-WT and eS7A-4KR cells determined by puromycin-treatment followed by western blotting analysis with anti-puromycin antibody. **b**, Quantification of levels of bulk translation in eS7A-WT and eS7A-4KR mutant cells during Tm treatment.

**
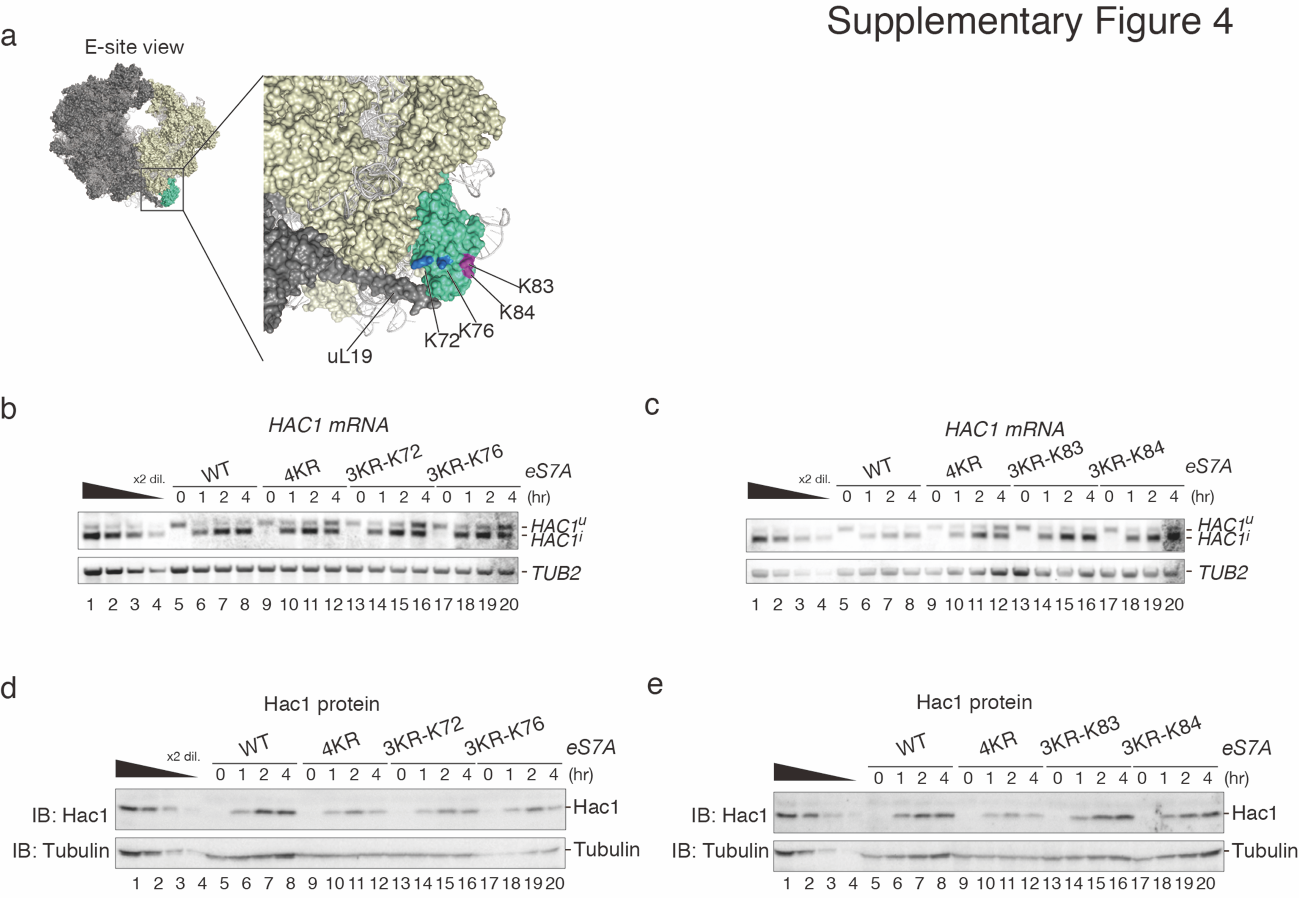
**

**Figure S4. Monoubiquitination of eS7A at lysine residue 83 or 84 is sufficient for splicing of *HAC1^u^* mRNA and Hac1 production. a**, Not4 potentially ubiquitinates four lysine residues on eS7A. **b-c**, Splicing of *HAC1*^u^ mRNA in four single-lysine eS7A mutant cells. **d-e**, Levels of Hac1 protein in four single-lysine eS7A mutant cells. All uncropped images are available in Supplemental Figure S11.

**
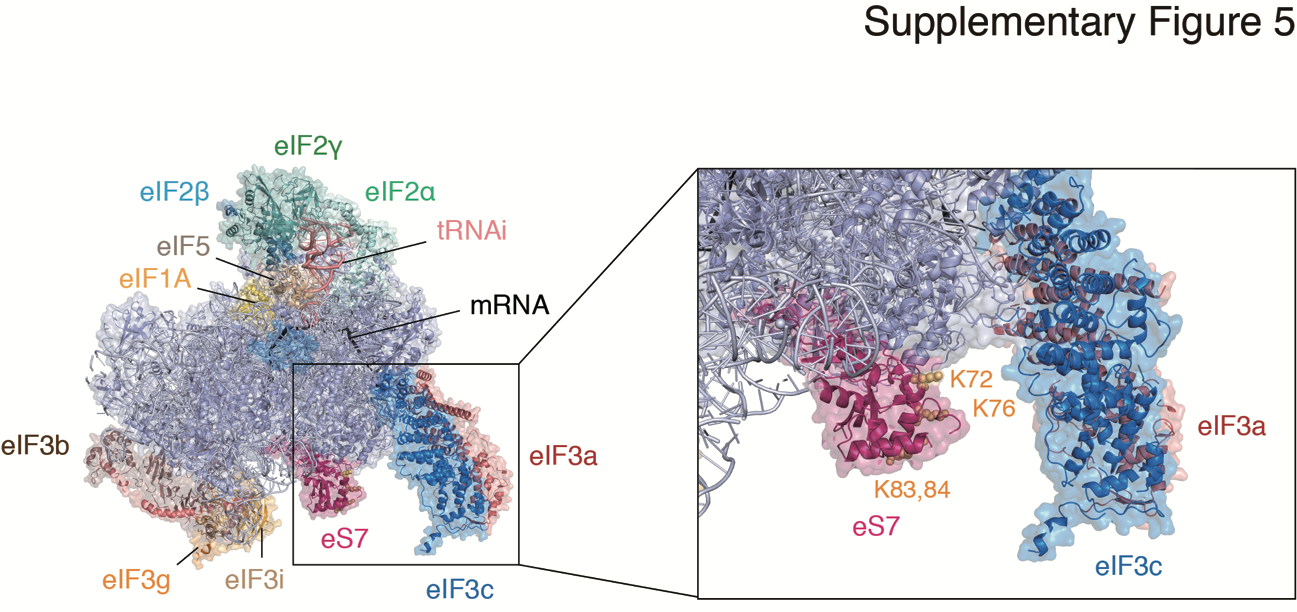
**

**Figure S5. Location of eS7 and the four ubiquitylation sites in a partial yeast 48S PIC model (adapted from Llacer et al., 2018; PDB accession code: 6FYY).** The 48S PIC containing Met-tRNA_i_^Met^ (salmonpink), eIF2α (light green), eIF2γ (green), eIF2β (teal), eIF5–NTD (light brown), eIF1A (yellow), eIF3a (red), eIF3b (brown), eIF3c (blue), eIF3i (yellow orange) and eIF3g (orange) is shown. eS7 is highlighted in hot pink, with its four lysine residues as ubiquitylation sites (K72, K76, K83, and K84) shown in orange spheres. The figure was generated using PyMOL (PyMOL Molecular Graphics System, Version 2.3.0. Schrodinger, LLC.).

**
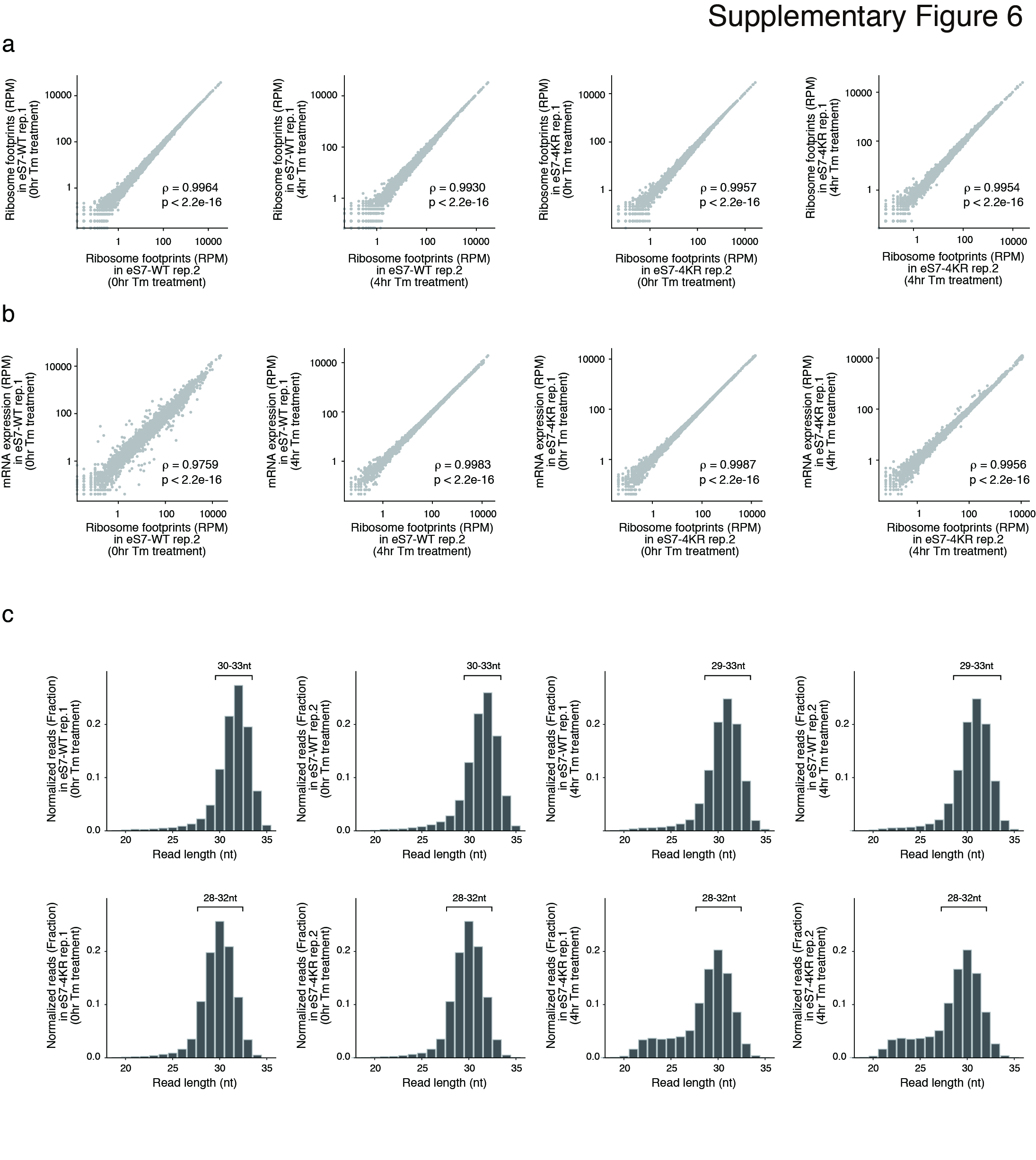
**

**Figure S6.** **Correlation between replicates of ribosome profiling.** **a**, Ribosome footprints in eS7A-WT replicates. **b**, mRNA expression in eS7A-WT replicates. **c**, Distribution of read lengths in eS7A-WT and eS7A-4KR replicates.

**
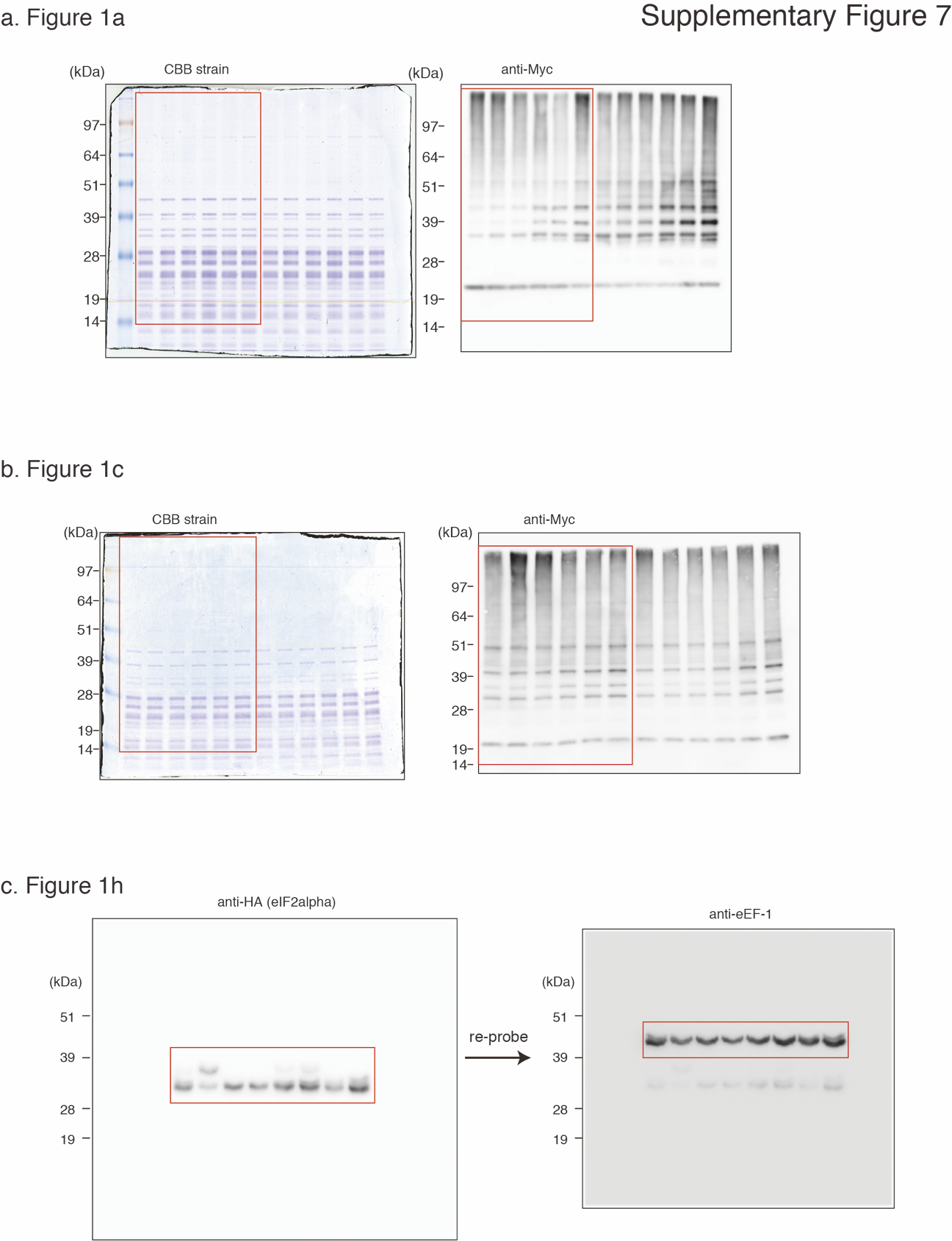
**

**Figure S7. Original gel image and blots of Figure 1.**

**a,** Original gel image and blots of Figure 1a. Ubiquitination of ribosome proteins was facilitated after Tm addition. **b**, Original gel image and blots of Figure 1c. Dependence of eS7A mono- and poly-ubiquitination on Not4. **c**, Original blots of Figure 1h. The phosphorylation levels of eIF2α in WT and S52A mutants during the amino acid starvation or the presence of Tm. All cropped regions were indicated by red line.

**
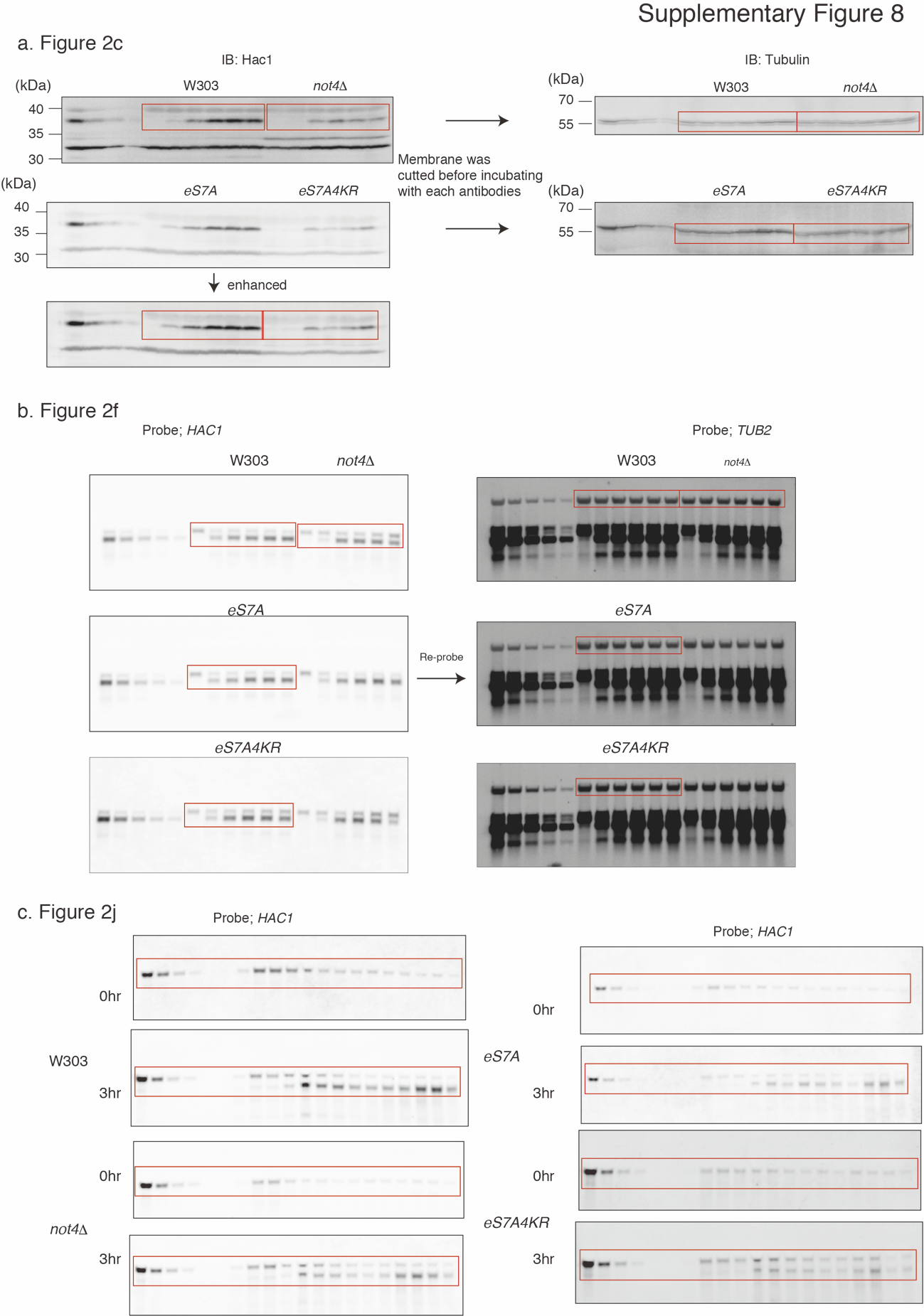
**

**Figure S8. Original images of western blots in Figure 2.**

**a**, Original images of western blots in Figure 2c. Western blots of Hac1 and tubulin protein expression after incubation with Tm (1 µg/mL) for 0 to 4 h. **b**, Original images of northern blots in Figure 2f. *HAC1* and *TUB2* mRNA expression after incubation with Tm (1 µg/mL) for 0 to 4 h. **c**, Original blots of northern blot in Figure 2j. Sucrose density gradient centrifugation followed by northern blotting was performed in W303, *not4*Δ, *eS7WT* and *eS7a-4KR* cells with or without Tm (1 µg/mL). All cropped regions were indicated by red line.

**
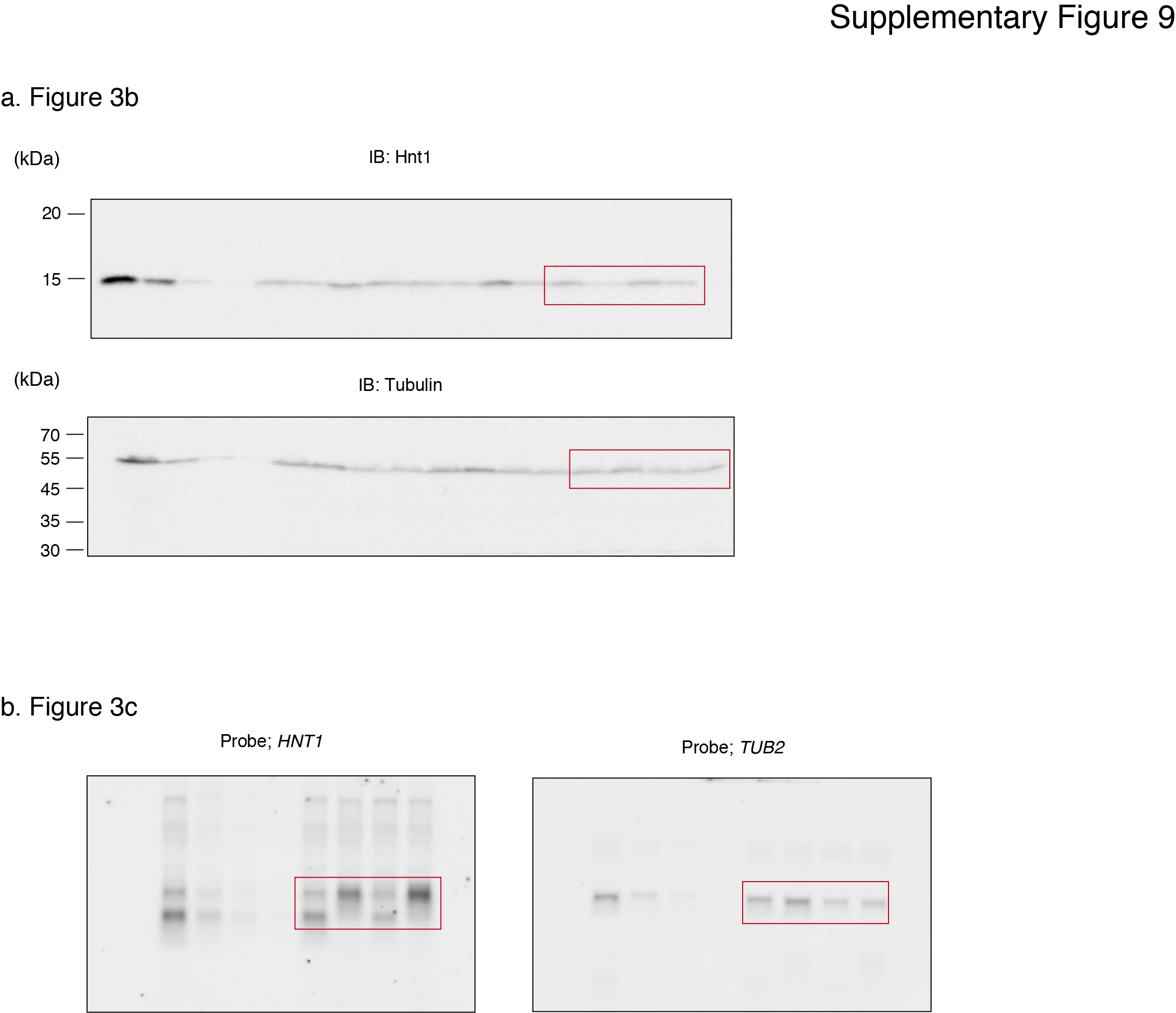
**

**Figure S9. Original images of western blots in Figure 3.**

**a,** Original images of western blots of Hnt1 and tubulin expression after incubation with Tm (1 µg/mL) for 0 to 4 h shown in Figure 3b. **b**, Original images of northern blots of *HNT1* and *TUB2* mRNA expression after incubation with Tm (1 µg/mL) for 0 to 4 h in Figure 3c. All cropped regions were indicated by red line.

**
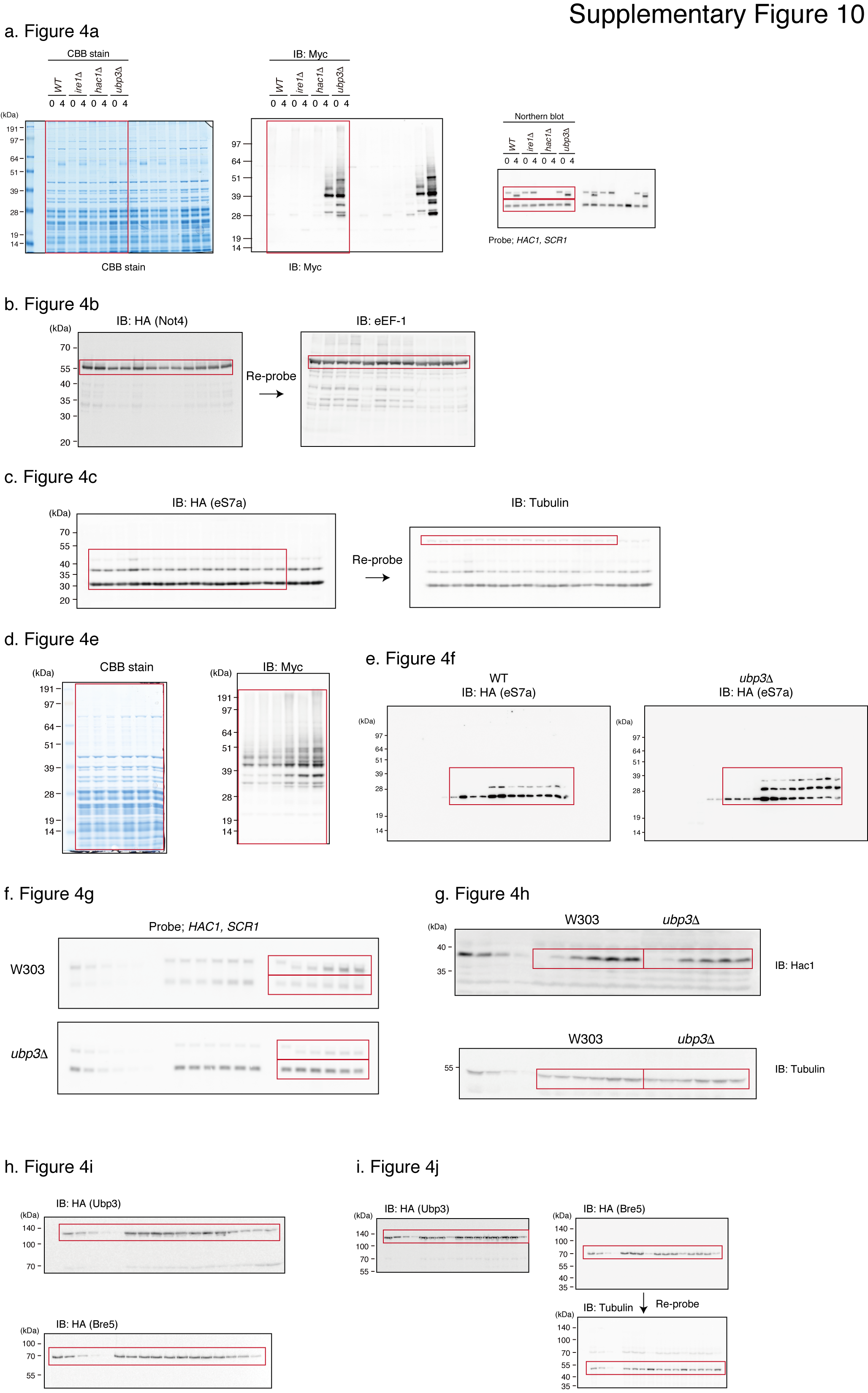
Figure S10. Original images of Figure 4**

**a**, Original images of Figure 4a. CBB stained gels and western blots of ribosome purified lysates from the indicated strains, and Northern blots of *HAC1* and *SCR1* mRNA expression. **b**, Original images of Figure 4b. Western blots of Not4-3HA and eEF-1 protein expression after incubation with Tm (1 µg/mL) for 0 to 4 h. **c**, Original images of Figure 4c. Western blots of eS7A-HA and tubulin protein expression after incubation with Tm (1 µg/mL) for 2 h.

**d**, Original images of Figure 4e. CBB stained gels and western blots of ribosome purified lysates from the *ubp3*Δ strain. **e**, Original images of Figure 4f. Western blots of samples fractionated by sucrose density gradient ultracentrifugation. **f**, Original images of northern blots in Figure 4g. *HAC1* and *TUB2* mRNA expression after incubation with Tm (1 µg/mL) for 0 to 4 h. **g**, Original images of western blots in Figure 4h. Western blots of Hac1 and tubulin protein expression after incubation with Tm (1 µg/mL) for 0 to 4 h. **h**, Original images of Figure 4i. Western blots of Ubp3-3HA, Bre5-3HA and tubulin protein expression after incubation with Tm (1 µg/mL) for 0 to 4 h. **i**, Original images of Figure 4j. Western blots of Ubp3-3HA, Bre5-3HA and tubulin protein expression in WT, *ire1Δ* and *hac1Δ* strains after incubation with Tm (1 µg/mL) for 0 to 4 h. All cropped regions were indicated by red line.

**
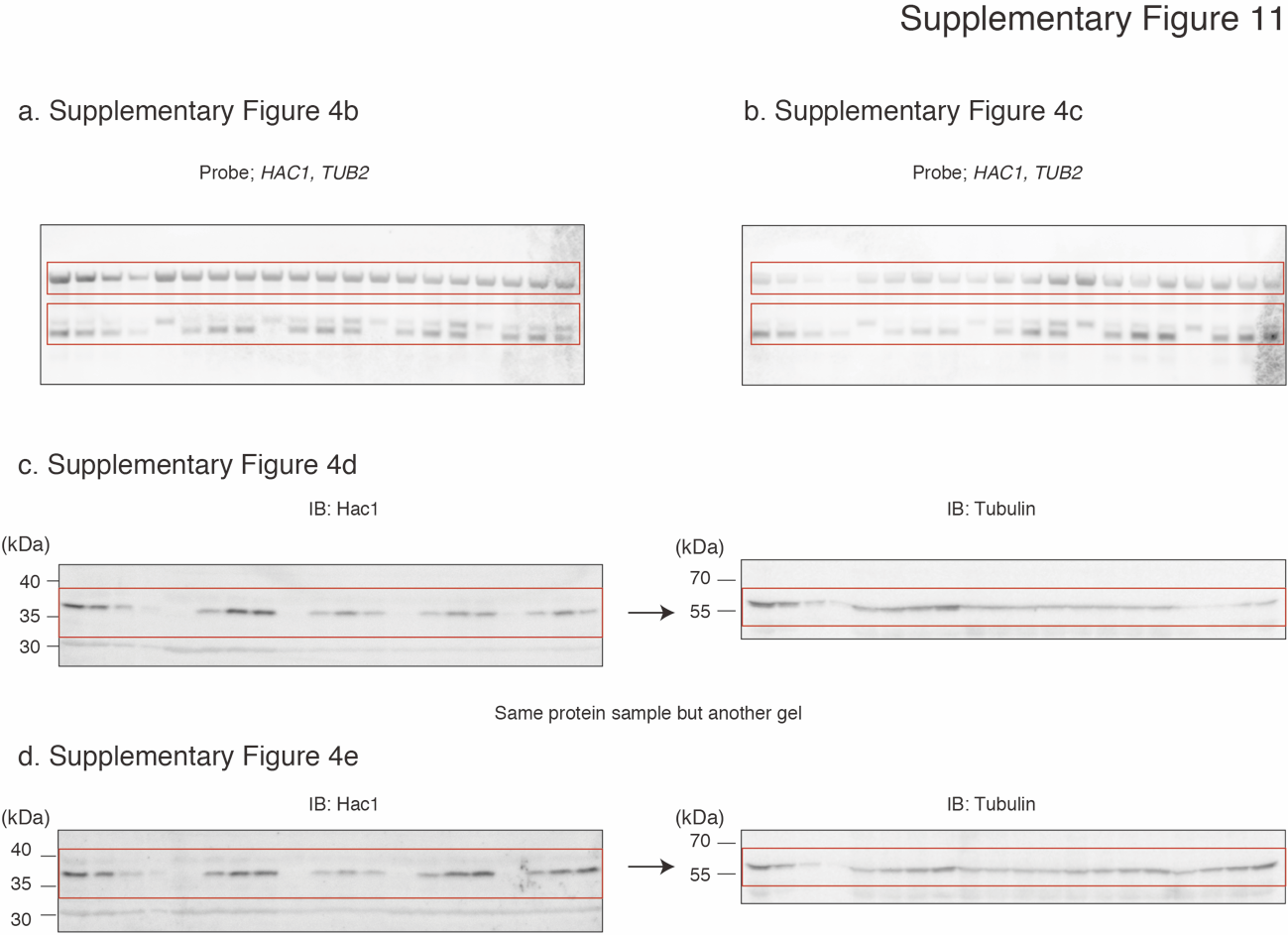
**

**Figure S11. Original images of Figure S4**

**a,b,** Original images of western blots of Hac1 and tubulin expression after incubation with Tm (1 µg/mL) for 0 to 4 h shown in Figure S4b,c. **c**,**d**, Original images of northern blots of *HAC1* and *TUB2* mRNA expression after incubation with Tm (1 µg/mL) for 0 to 4 h in Figure S4d,e. All cropped regions were indicated by red line.

**References**

1. Inada, T., Winstall, E., Tarun, S.Z., Jr., Yates, J.R., 3rd, Schieltz, D. and Sachs, A.B. (2002) One-step affinity purification of the yeast ribosome and its associated proteins and mRNAs. *RNA*, **8**, 948-958.

2. Matsuo, Y., Ikeuchi, K., Saeki, Y., Iwasaki, S., Schmidt, T.C., Udagawa, T., Sato, F., Tsuchiya, H., Becker, T., Tanaka, K. *et al.* (2017) Ubiquitination of Stalled Ribosome Triggers Ribosome-associated Quality Control. *Nature Communications*.
